# First report of B chromosomes in three neotropical thorny catfishes (Siluriformes, Doradidae)

**DOI:** 10.3897/CompCytogen.v11i1.10496

**Published:** 2017-01-09

**Authors:** Fábio Hiroshi Takagui, Ana Lucia Dias, José Luís Olivan Birindelli, Ana Claudia Swarça, Renata da Rosa, Roberto Laridondo Lui, Alberto Sergio Fenocchio, Lucia Giuliano-Caetano

**Affiliations:** 1 Laboratory of Animal Cytogenetics; Department of General Biology, CCB, Universidade Estadual de Londrina. Rodovia Celso Garcia Cid, PR 445, km 380, Londrina-Brasil; 2 Museum of Zoology, Department of Animal and Plant Biology, CCB, Universidade Estadual de Londrina. Rodovia Celso Garcia Cid, PR 445, km 380, Londrina-Brasil; 3 Laboratory of Histology and Genetics; Department of Histology; Center of Biological Sciences (CCB); Universidade Estadual de Londrina (UEL). Londrina-Brasil; 4 Laboratory of Cytogenetics; Center of Biological Sciences and Health: Universidade do Oeste do Paraná, Campus Cascavel. Cascavel - Brasil; 5 Laboratory of General Cytogenetics; Department of Genetics; Facultad de Ciencias Naturales; Universidad Nacional de Misiones. Posadas- Argentina

**Keywords:** Centric fusion, chromosomal rearrangements, diploid number, neotropical fish, pericentric inversions, supernumerary chromosome

## Abstract

The family Doradidae (Siluriformes) is an important group of fishes endemic to freshwater ecosystems in South America. Some cytogenetic studies have been conducted focused on the group; however, there are no reports on the occurrence of B chromosomes for the family. In this paper the chromosomal characteristics of *Platydoras armatulus* (Valenciennes, 1840), *Pterodoras granulosus* (Valenciennes, 1821) and *Ossancora punctata* (Kner, 1855) were investigated through classical cytogenetics approaches. The conventional staining reveals 2n=58 in *Platydoras armatulus* and *Pterodoras granulosus*, however with distinct karyotypic formulae, possibly originated by pericentric inversions. In *Ossancora punctata* a derivate karyotype was described with 2n=66 and predominance of acrocentric chromosomes. The C banding pattern was resolutive in discriminating the three species, being considered an important cytotaxonomic marker. All species showed B chromosomes totally heterochromatic with non-Mendelian segregation during meiosis and low frequencies in mitotic cells. The probably origin of these additional elements was through fragmentations of chromosomes of the standard complement, which occurred recently and independently in these three species. The diploid number observed in *Ossancora punctata* is an evidence of centric fusions and up to the moment it is the highest diploid number reported for Doradidae.

## Introduction

Cytogenetic studies in Doradidae are scarce and restricted to nine species. Eight of these bear 58 chromosomes and single nucleolus organizer regions (NORs) in terminal positions (Eler et al. 2007, Milhomem et al. 2008). The exception to this pattern is *Trachydoras paraguayensis* (Eigenmann & Ward, 1907) with 56 chromosomes and single NORs in an interstitial position (Fenocchio et al. 1993, Baumgärtner et al. 2016). The members of Doradidae are popularly named thorny catfish and comprise 94 species and 33 genera (Froese and Pauly 2016) endemic to freshwater ecosystems in South America. The family is easily diagnosed among catfishes by the presence of a row of bony midlateral scutes, each usually bearing a single thorn (Birindelli 2014). Phylogenetic studies based on molecular (Moyer et al. 2004, Arce et al. 2013) and morphological (Birindelli 2014) data support the monophyly of Doradidae.

Supernumerary chromosomes have already been reported in several neotropical Siluriformes families, however up to now they have not been observed in Doradidae (Carvalho et al. 2008, Lui et al. 2009). These additional elements were described in different organisms and can originate in two ways: from chromosomal rearrangements in chromosomes from the A complement (the most common), or as a consequence of interspecific crosses. Regardless of their origin, the majority of B chromosomes due to not possess genes and follow an independent evolutionary path characterized by structural differentiation mechanisms, including the accumulation of different repetitive DNA sequences (Camacho et al. 2000).

In most organisms, the B chromosomes are dispensable elements, as their presence is not associated with phenotypic alterations. However, there are exceptions, as described in *Nectria haematococca* Samuels & Rossman, 1999 where the Bs possess resistance genes which grant a better pathogenicity (Coleman et al. 2009), and in *Lithochromis rubripinnis* Seehausen, 1998 in which B chromosomes have a functional effect in sex determination (Yoshida et al. 2011). According to Valente et al. (2016), the recent development of molecular biology associated with the advances in next-generation sequencing technologies have increased knowledge about the biological importance of B chromosomes, revealing that the presence of many genes and other transcriptionally active sequences can modulate the activity of autosomal genes.

In the present study, the karyotypic structure of *Platydoras armatulus*, *Pterodoras granulosus* and *Ossancora punctata* was investigated in mitotic and meiotic cells. This comparative analysis to provide a better understanding of the karyotype diversification in Doradidae, reporting for the first time the occurrence of B chromosomes and discussing the probably origins of this feature in this family.

## Material and methods

In the present study, cytogenetic analyses were performed on 9 females and 8 males of *Platydoras armatulus*; 3 females and 6 males of *Ossancora punctata*, all collected in the Miranda river, in Corumbá, Mato Grosso do Sul, in the Brazilian Pantanal (19°31'25"S 57°02'26"W). Additionally, 5 females and 4 males of *Pterodoras granulosus* also collected in the Paraná river, in Pauliceia, São Paulo, Brazil (21°06'10.26"S 51°47'14.1"W), were analyzed. The collection of specimens was authorized by ICMBio (Instituto Chico Mendes de Conservação da Biodiversidade). After processing and subsequent fixation of the material, all specimens were deposited in the Museu de Zoologia da Universidade Estadual de Londrina (data available via SpeciesLink).

Before euthanasia (48 hours), the specimens received an intraperitoneal injection of 2 ml of Broncho-vaxom (bacterial lysate) to trigger an inflammatory process and hence increase the number of kidney cells in mitotic division (Molina et al. 2010). After this, all specimens were anesthetized with clove oil (eugenol) and sacrificed to obtain the mitotic chromosomes from kidney cells (Bertollo et al. 1978) and meiotic chromosomes from testis cells (Kligerman and Bloom 1977). The metaphasic chromosomes were classified as metacentric, submetacentric, subtelocentric and acrocentric according to ratio of arms proposed by Levan et al. (1964). The heterochromatin pattern was determined using the C-banding technique (Sumner 1972) with a modification in staining phase (Lui et al. 2012).

## Results

### *Platydoras armatulus*

**Figure 1.**
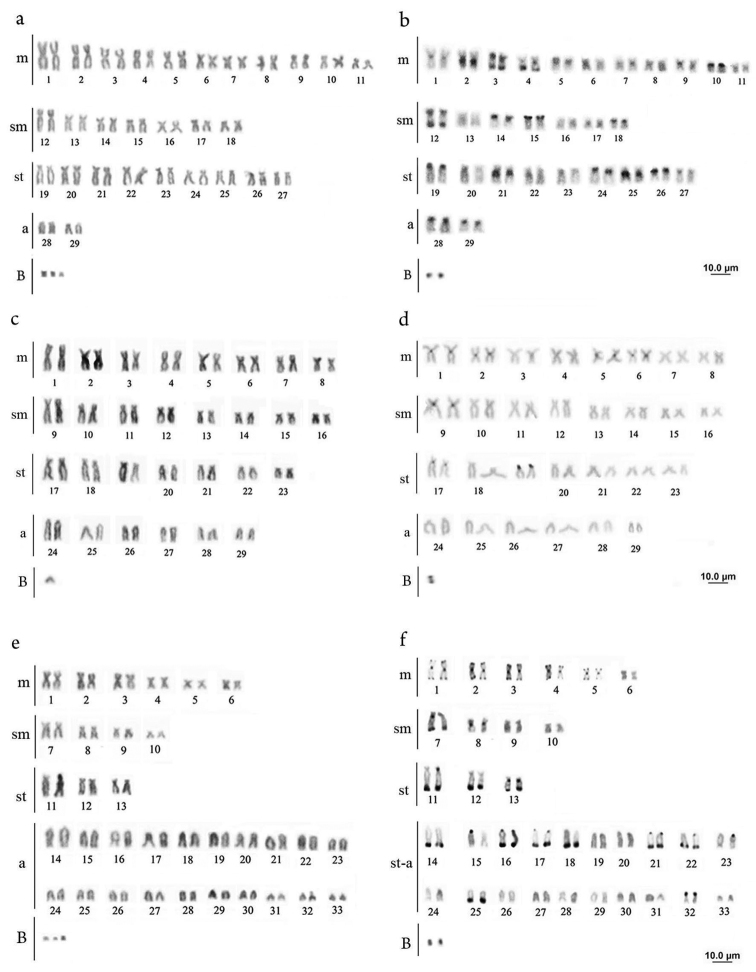
Karyotypes after conventional staining and C-banding – *Platydoras armatulus* **a** Giemsa staining reveals 2n=58 (22m+14sm+18st+4a) and 1-3 B chromosomes **b** C-banding pattern characterized by the many heterochromatin blocks in different positions, including in B chromosomes. *Pterodoras granulosus* **c** Giemsa staining also reveals 2n=58 but with distinct karyotypic formulae: 16m+16sm+14st+12a and 1 B chromosome **d** a few heterochromatin blocks was evidenced after C banding, observe the B chromosome totally heterochromatic. *Ossancora punctata* **e** After Giemsa staining it was observed 2n=66 (12m+8sm+6st+40a), the high number of subtelocentric and acrocentric chromosomes is a remarkable feature of this specie **f** C-banding reveals heterochromatin regions in terminal position and in B chromosomes.

**Figure 2.**
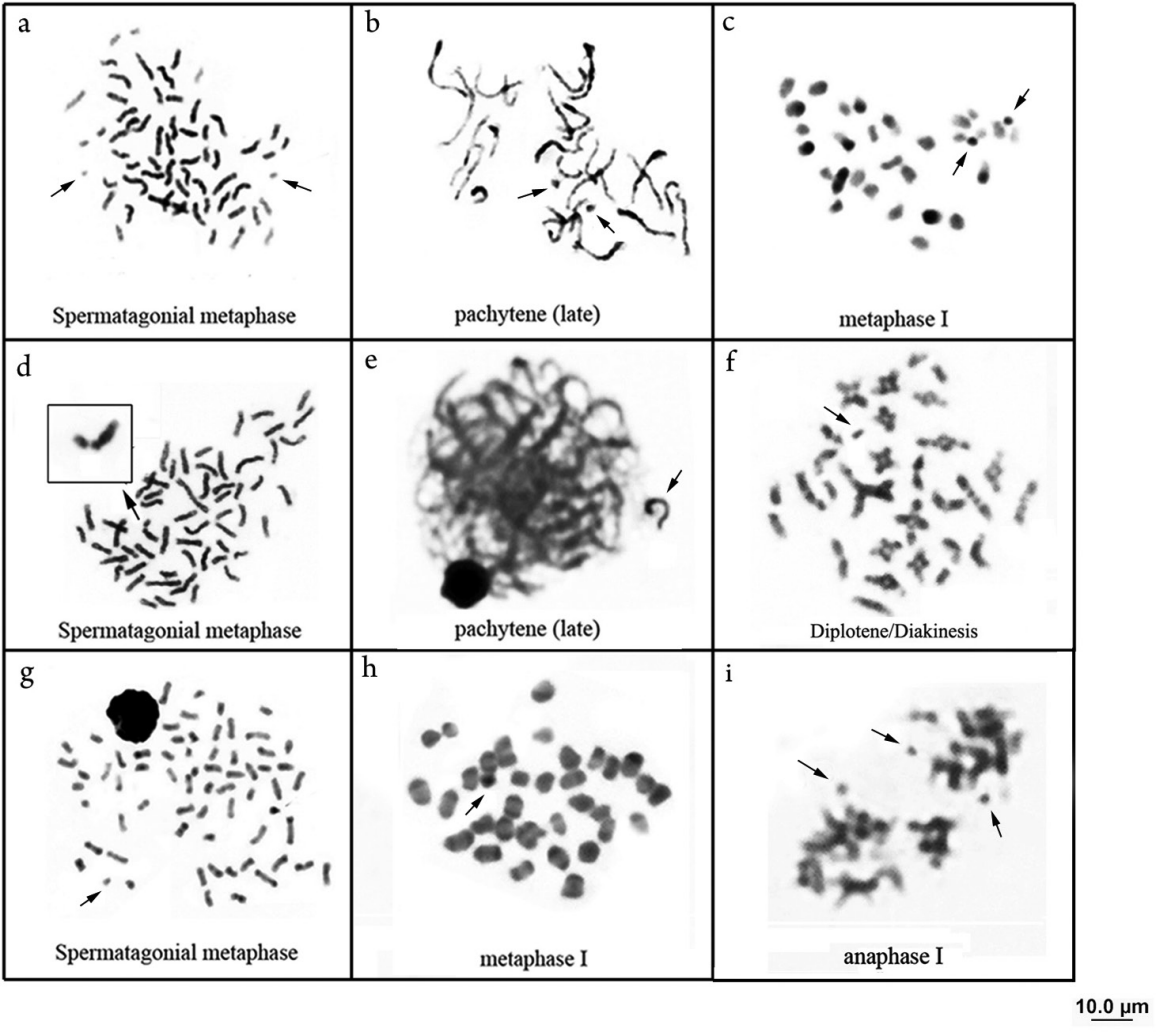
Meiotic cells in different phases with B chromosomes evidenced after C-banding - *Platydoras armatulus* **a** spermatogonial metaphase with 58 chromosomes and 2 B chromosomes **b** late pachytene with bivalents in advanced condensation stage, note two B chromosomes forming univalents without homologies of standard complement **c** metaphase I with 27 bivalents and two B chromosomes. *Pterodoras granulosus* **d** spermatogonial metaphase composed by 58 chromosomes and one B chromosome **e** late pachytene, the isolated univalent probably correspond to B chromosome **f** diplotene/diakinesis with 27 bivalents and one B chromosome, note the high number of chiasms. *Ossancora punctata* **g** spermatogonial metaphase with 66 chromosomes and one B chromosome **h** metaphase I reveals heterochromatic B chromosome and 33 bivalents **i** anaphase I, observe the late segregation of B chromosomes.

All specimens analyzed exhibited 58 chromosomes (22m + 14sm + 18st + 4a). Eleven samples showed cells carrying from 1-3 B chromosomes (Fig. 1a) with interindividual frequencies ranging from 5.25% to 61.90% (Table 1). C-banding evidenced heterochromatin blocks in the pericentromeric and terminal regions in the short arm of pairs 3, 5, 10, 12, 14, 15, 18, 19, 24, 26, 28 and in the long arm of pairs 3, 4, 12. Interstitial heterochromatin regions also occurred in pairs 2, 21 and 25. The B chromosomes are totally heterochromatic (Fig. 1b). C-banding applied to meiotic cells confirmed the results observed in mitosis in: spermatogonial metaphase with 58 chromosomes (Fig. 2a); late pachytene (more condensed) (Fig. 2b) and metaphase I, with 29 bivalents (Fig. 2c).

**Table 1.** Frequencies of supernumerary chromosomes in *Platydoras armatulus*, *Pterodoras granulosus* and *Ossancora punctata*. ♀= female; ♂= male.

Species/Samples	Sex	Number of B/cell				Total of cells	Cells with B
		0	1	2	3		
***Platydoras armatulus***							
**4156**	♂	17	6	8	4	35	**51.42%**
**4157**	♀	29	4	0	0	33	**12.12%**
**4158**	♂	33	6	3	2	43	**23.25%**
**4159**	♀	14	0	0	0	14	**0%**
**4160**	♂	16	8	12	6	42	**61.90%**
**4161**	♂	17	7	10	5	39	**56.41%**
**5032**	♂	22	4	1	0	27	**18.51%**
**5320**	♀	10	0	0	0	10	**0%**
**5322**	♀	18	1	0	0	19	**5.26%**
**5325**	♀	15	0	0	0	15	**0%**
**7**	♂	22	3	2	1	28	**21.42%**
**8**	♂	31	1	0	2	34	**8.82%**
**9**	♀	25	0	0	0	25	**0%**
**80**	♂	11	1	3	0	15	**26.66%**
**81**	♀	19	0	0	0	19	**0%**
**82**	♀	21	0	1	1	23	**8.69%**
**83**	♀	28	0	0	0	28	**0%**
***Pterodoras granulosus***							
**601**	♀	16	2	0	0	18	**12.5%**
**602**	♀	15	3	0	0	18	**20%**
**603**	♂	54	9	0	0	63	**16.6%**
**604**	♀	63	15	0	0	78	**23.8%**
**617**	♂	32	4	0	0	36	**12.5%**
**618**	♀	21	0	0	0	21	**0%**
**619**	♀	23	0	0	0	23	**0%**
**628**	♂	16	0	0	0	16	**0%**
**631**	♂	35	6	0	0	41	**17.1%**
***Ossancora punctata***							
**4561**	♀	25	5	3	1	34	**26.47%**
**4566**	♂	23	7	2	4	36	**36.11%**
**5119**	♂	21	4	2	3	30	**30%**
**5120**	♂	32	0	3	2	37	**13.51%**
**5692**	♂	31	1	0	0	32	**3.12%**
**5694**	♀	14	0	0	0	14	**0%**
**5695**	♀	6	0	0	0	6	**0%**
**5696**	♂	23	1	0	0	24	**4.16%**
**5332**	♂	32	3	0	4	39	**17.94%**

### *Pterodoras granulosus*

Conventional staining with Giemsa revealed 58 chromosomes, with a karyotype formula 16m + 16sm + 14st + 12a. In six specimens, one acrocentric supernumerary chromosome was observed (Fig. 1c) with interindividual frequencies ranging from 12.5% to 23.8% (Table 1). C-banding identified few blocks of heterochromatin restricted in some centromeres, short arm of pair 19 and in B chromosome (Fig. 1d). In meiotic analyses the B chromosomes were observed totally heterochromatic in: spermatogonial metaphase (Fig. 2d), late pachytene with the B chromosome isolated (Fig. 2e) and diplotene/diakinesis (Fig. 2f).

### *Ossancora punctata*

The studied specimens presented 66 chromosomes and a karyotype formula of 12m + 8sm + 6st + 40a. Among all nine specimens analyzed, seven exhibited B chromosomes (Fig. 1e), and the frequencies were considered low, ranging from 3.12 % to 36.11 % of the specimen cells (Table 1). C-banding revealed pericentromeric heterochromatin in most chromosomes, as well as terminal blocks on the long arm of subtelo-acrocentric chromosomes and at the both ends of most metacentric chromosomes (Fig. 1f). The microchromosomes also presented themselves entirely heterochromatic in meiotic analyses in: spermatogonial metaphase (Fig. 2g), metaphase I with 33 bivalents (Fig. 2h) and anaphase (Fig. 2i).

## Discussion

Phylogenetic analysis based on morphological and molecular data supports the monophyly of Doradidae that, together with Auchenipteridae, constitutes the superorder Doradoidea (Moyer et al. 2004, Arce et al. 2013, Birindelli 2014). According to some authors, the ancestor of Doradidae had a karyotype composed by 58 chromosomes (Eler et al. 2007, Milhomem et al. 2008, Baumgärtner et al. 2016). In fact, this diploid number is present in *Wertheimeria maculata* Steindachner, 1877 (Eler et al. 2007) the species considered, with *Kalyptodoras bahiensis*, the sister group of this family (Birindelli 2014).

Notwithstanding, not all doradid species have 2n = 58, as is the case of *Trachydoras paraguayensis* with 2n = 56 chromosomes (Fenocchio et al. 1993, Baumgärtner et al. 2016) and *Ossancora punctata* with 2n = 66 (present study). Baumgärtner et al. (2016) identify interstitial telomeric sequences (ITS) in *Trachydoras paraguayensis*, demonstrating the emergence of the 2n = 56 from a karyotype with 58 chromosomes by centric fusion. Diversely, in *Ossancora punctata* the 2n = 66 is the largest diploid number ever reported for the family and probably originated due to centric fissions resulting in a karyotype with many subtelocentric and acrocentric chromosomes. These variations in diploid numbers show that the pericentric inversions are not the only chromosomal rearrangements that generate macro-structural variability (Eler et al. 2007, Milhomem et al. 2008).

The dispersion of heterochromatic regions is a high variable in Doradidae. *Pterodoras granulosus* exhibited few blocks, similarly described for *Platydoras costatus* Linnaeus, 1758 (Milhomem et al. 2008), but distinct from the pattern observed in *Platydoras armatulus* which exhibited many chromosomes bearing heterochromatin blocks in terminal and interstitial positions. This divergence observed in *Platydoras* can be an excellent cytogenetic marker, because these two species have 58 chromosomes and similar karyotypic formulae. The heterochromatin pattern of *Ossancora punctata* is similar to that described in *Hassar wilderi* Kindle, 1895 (Eler et al. 2007), *Hassar orestis* Steindachner, 1875, *Hassar* sp. and *Tenellus ternetzi* Eigenmann, 1925 (Milhomem et al. 2008) with many terminal blocks, some of these located in both chromosome arms. This C-band pattern reinforced the phylogenetic proximity between these three genera, which constitute one of the most derived clades of Doradidae (Birindelli 2014).

Cytogenetic studies in Neotropical Siluriformes revealed the occurrence of B chromosomes in more than 25 species, including representatives of the families Heptapteridae, Callichthyidae, Pimelodidae, Pseudopimelodidae, Auchenipteridae, Tricomycteridae and Loricariidae (Lui et al. 2009). The B chromosomes of *Platydoras armatulus*, *Pterodoras granulosus* and *Ossancora punctata* presented similar structural characteristics, even though the frequencies in mitotic cells were highly variable. This numerical variability is an evidence of the non-Mendelian segregation theory proposed by Jones and Rees (1982) and occurs because the B chromosomes possess a delayed migration during anaphase, as can be observed in some germ cells of *Ossancora punctata* (Fig. 2i). Another feature visualized in some spermatocytes was the presence of B chromosomes forming a univalent isolated of the standard complement. This meiotic behavior suggested a structural differentiation of B chromosomes in relation to the standard complement due to accumulation of different families from repetitive DNA (Camacho et al. 2000).

In Neotropical fish, the mechanisms responsible for the origin and evolution of B chromosomes remain unclear, as several theories were proposed (Lui et al. 2009, Blanco et al. 2012, and others). The B microchromosomes were described in distinct neotropical fishes, including *Schizodon* Agassiz, 1829, *Astyanax* Baird et Girard, 1854, *Moenkhausia* Eigenmann, 1903, *Cyphocharax* Fowler, 1906, *Steindacnerina* Fowler, 1906, *Prochilodus* Agassiz, 1829, *Rhamdia*, Bleeker, 1858 *Iheringichthys* Eigenmann et Norris, 1900, *Callichthys* Scopoli, 1777, *Megalonema* Eigenmann, 1912, *Pimelodella*, Eigenmann et Eigenmann, 1888 and *Loricaria* Linnaeus, 1758 (Carvalho et al. 2008, Lui et al. 2009). An interesting hypothesis to explain the origin of these additional genomic elements is the fragmentation in standard karyotype (Sampaio et al. 2015). Considering the morphological type, non-Mendelian segregation and low frequencies in mitotic cells, it seems likely that the B chromosomes observed in *Platydoras armatulus*, *Pterodoras granulosus* and *Ossancora punctata* have a recent origin from fragmentation in chromosomes from A complement.

This study contributed with relevant information to the better understanding of the karyotype variability in Doradidae. In this family, the 2n=58 is considered a primitive condition, such that the chromosomal diversification is based primarily on pericentric inversions and at lower frequency fissions and fusions. Additionally, the mitotic and meiotic analysis revealed at the first time in Doradidae the occurrence of B chromosomes, which originated recently from fragmentations in chromosomes of standard complement. Additional studies such as the isolation and molecular characterization of these chromosomes can be resolutive in confirming its origin and evolution.
